# Analysis of Sports Supplements Consumption in Young Spanish Elite Dinghy Sailors

**DOI:** 10.3390/nu12040993

**Published:** 2020-04-03

**Authors:** Israel Caraballo, Raúl Domínguez, Eduardo J. Guerra-Hernandez, Antonio J. Sánchez-Oliver

**Affiliations:** 1 GALENO Research Group, Department of Physical Education, Faculty of Education Sciences, University of Cádiz, 11519 Puerto Real, Spain; israel.caraballo@uca.es; 2 Biomedical Research and Innovation Institute of Cádiz (INiBICA) Research Unit, 11519 Cádiz, Spain; 3 Collegue of Health Sciences of Isabel I University, Isabel I University, 09004 Burgos, Spain; 4 Department of Nutrition and Food Science, University of Granada, 18071 Granada, Spain; ejguerra@ugr.es; 5 Department of Human Motricity and Sports Performance, University of Seville, 41013 Seville, Spain; sanchezoliver@us.es

**Keywords:** sports performance, nutrition, hydration, ergogenic aids, nautical sport

## Abstract

The sports performance of dinghy sailors is determined by their state of nutrition and hydration. Sports supplementation plays a prominent role in elite sailors, being essential in periods of competition due to its characteristics. This study aims to analyze the consumption of sports supplements (SS) in the different categories and groups of sailors based on the level of evidence, differentiating according to sex, competitive level, and type of boat. A total of 42 sailors from national and international levels and belonging to the Laser, 420, Techno-293 and RS:X classes participated in this study. They completed a questionnaire with questions about the consumption of SS and the possible repercussions on health and/or sports performance. The results were analyzed based on the different categorizations and group organization recently established by the Australian Institute of Sport (AIS), as well as by sex, level of competition and class to which the participants belonged. The male sailors and those who competed internationally had a higher prevalence in the consumption of SS. Among the classes of vessels studied, class 420 had the lowest SS consumption. SS intake was higher during competition days, regardless of sex or level of competition. Based on the classification established by the AIS, statistically significant differences were observed in sex, level of competition, and the type of boat.

## 1. Introduction

Sailing is a physical activity with certain peculiarities compared to other sports since performance will not only depend on the physical condition of the sailor, but it can also be affected by other variables, such as the characteristics of the boat and the meteorological conditions [1,2,3]. Thus, the physical, technical, and tactical requirements that the sailor must face during navigation will be very specific and may present very notable variations [4].

The term dinghy sailing refers to those sports sailing boats in which the weight of the boat and the weight of the sailor are not significantly different [5,6]. The main objective of this class is to keep the boat at its maximum speed to make the route established in the shortest time possible [4]. This class is characterized by the action of a hiking bench. It is the technical gesture used by the sailor to keep the boat stable. In this action, the sailor takes his body out of the boat to use it as a lever arm while keeping his feet fixed on a strap located in the center of the boat. This specific technical gesture is carried out by the sailor to avoid the lateral inclination of the boat by pulling his/her body out of it, and in which the sailor spends up to 94% of the total sailing time [7]. The type of muscular action that is carried out in this gesture is defined by the term “quasi-isometric” since there are small and constant modifications in the articular range in order to adapt to the changes that occur in the boat during the navigation [8]. Studies on energy metabolism in dinghy sailors are mainly focused on the action of hiking bench, showing maximum oxygen consumptions (VO_2max_) in the range of 35–45% VO_2max_ [6,9,10]. The most important factors in the performance of the sailor are the muscular endurance of the main musculature involved in the hiking action and the maximum isometric strength of the quadriceps [11,12]. Some studies have shown that the level of aerobic capacity in elite sailors is like that of athletes from other sports disciplines [6,13]. In this sport, the demand for aerobic capacity will be conditioned by the wind speed and will have a linear relationship; thus, the greater the wind speed, the greater the aerobic demand, reaching values of 80–90% VO_2max_ when the wind speed reaches 20 knots [9,14].

The fitness level of the sailor is one of the main factors related to performance [15], and it is influenced by numerous variables, such as sex, age, nutrition, and hydration. Nutrition and hydration, in addition to influencing the physical condition of the sailor, are presented as limiting factors of utmost importance in sailing performance [4,16,17,18]. Sailing is a physically challenging sport in which the regattas are characterized by having a duration of several hours in a row for more than one day of competition, taking place at daylight hours and in seasons of the year with high temperatures and humidity [19]. Moreover, the competition rules and the small size of the boat allow the sailor to carry small amounts of food and drinks [16]. Consequently, it is likely that the nutritional status and hydration of sailors are compromised during the competition; thus the replenishment of nutrients and hydration becomes fundamental during pre-competition, competition, and post-competition periods [20]. Despite this, there are no specific nutrition guidelines for this sport, and the literature on the intake of food and liquids of sailors before, during, and after the regattas is scarce, although existing studies reveal that nutritional practices of sailors do not meet current nutrition guidelines [16].

When the level of competition increases significantly, an adequate intake of energy and nutrients becomes more important, since any benefit acquired, however small, can provide an advantage during the competition [21,22]. This possibility of improvement encourages athletes to consider the consumption of sports supplements (SS). The consumption of SS and its importance in the world of sports nutrition has grown significantly in recent years [23]. Although its use is widespread in the world of sports and despite the numerous SS there are in the sports world [24], only a few SS have been shown to improve sports performance, such as sports foods (they provide energy and nutrients when it is impractical to consume everyday foods, such as sports drinks, bars, and gels or whey protein), medical supplements (indicated for clinical alterations such as nutritional deficiency) and performance supplements (e.g., β-alanine, sodium bicarbonate, caffeine, creatine and nitrate, or beetroot juice) [25]. Due to the characteristics of the dinghy, some of the supplements with scientific evidence could be considered.

In any case, even in SS with considerable scientific evidence on performance improvement, ergogenic effects are subject to the individual, sport modality, and context [21,26]. Previous research has proclaimed the differences in SS consumption between men and women and between national and international athletes or between different sports modalities [21,24,27]. The inclusion of sports supplements could be recommended within the competitive nutrition plans of sailors for increasing training adaptations, during the competitions, or for preventing nutritional deficits. The only study about SS consumption in elite sailors indicates that 77% of its participants consumed some type of supplementation, and 38% did so on a regular basis. The most frequently used SS in this study were vitamins and minerals, proteins (amino acids), isotonic drinks, and energy bars [13].

Given the important role that hydration and nutrition play in performance in the light sailing class and, in the absence of studies that have assessed the prevalence of SS consumption, the objective of the present study is to analyze the consumption of SS based on the different categories and groups according to the level of evidence established by the Australian Institute of Sport (AIS) 2019 [25] in sailors, as well as the possible differences in sex, competitive level, and type of boat.

## 2. Materials and Methods

### 2.1. Participants

For recruiting the sample, the Andalusian Sailing Federation and clubs who participated in the official and international competition called XV New Year’s Race was contacted. In addition, as the athletes were minors (12–17 years old), their parents were contacted, and informed consent and authorization were sent for their record. Finally, of the 113 enrolled, 42 sailors (31 men and 11 women; 32 nationally and 10 internationally) of different classes—Laser class (*n =* 15), class 420 (*n =* 6), Techno-293 class (*n =* 12), and RS:X class (*n =* 9)—participated in this study. The boats in the Laser and 420 classes differ mainly from the Techno-293 and RS:X classes in the way these boats are manned. In the first two, the sailor is sitting, while in the other two he is standing. In addition, the sail in the Laser and 420 classes is fixed on the boat, and in the Techno-293 and RS:X classes, it is mobile. These last two classes are in the wind-surfing group. This investigation was approved by the Ethics Committee of the Alfonso X El Sabio University.

### 2.2. Procedure

The sailors who, voluntarily and with the prior consent of the parents, agreed to participate in this study, completed a survey on the consumption of SS during the XV New Year’s Race held between 27 and 30 December 2018 in El Port of Santa María (Cádiz, Spain). For the collection of data, a room was set up at the headquarters of the Andalusian Sailing Federation, and a schedule was established for sailors to attend the questionnaires. Previously, and to facilitate the process, the shifts to access the room were completed with the trainers.

### 2.3. Instruments

The questionnaire used was previously validated, assessing the validity of its content, indicating (i) its capacity to measure what it was created for, (ii) its application, analyzing benefits and defects, reviewing the instructions of the instrument, (iii) its structure, reviewing the formulation of the questions, the proposed sequence and the response scale, and (iv) its presentation, in which the best characteristics of the appearance and format of the instrument were identified [27]. This instrument has been used in different studies that have analyzed the consumption of SS in different sport modalities [22,23,26,27,28,29,30,31,32]. In addition, in previous studies, this survey achieved 54% of methodological quality [24], in which only 57 of the 164 questionnaires reviewed were approved. The questionnaire is organized in three parts: (i) one in which the anthropometric, personal, and social data of the respondent are collected, (ii) a second one that aims to analyze sports activity and its contextualization, and (iii) another part related to SS consumption and its possible repercussions on health and/or sports performance. About the use of SS, the questionnaire recorded the SS at the moment of the completion on the questionnaire and consumption in general. This questionnaire includes questions relatives to the training period of the consume (training period, competitive period, training and competitive period, transition period, always or never), the timing relative to the effort (before, during, and after training, or indifferently). Also, subjects were asked about the consumption of banned substances and relatives to their felt about the use of banned substances in their sport.

### 2.4. Statistical Analysis

The normality of the variables was confirmed by a Kolmogorov–Smirnoff test, while homoscedasticity was verified by means of Levene’s test. For the analysis of the differences in SS consumption in the different categories and groups based on the level of evidence established by the AIS (2019) [25] by sex and between competition levels (national vs. international), a Student *t*-test for independent samples was applied and Cohen’s d effect size was calculated [33]. Effect sizes (ES) were considered as large (above 0.8), moderate (between 0.8 and 0.5), small (between 0.5 and 0.2), and trivial (lower than 0.2). In addition, in those SS that determined a prevalence in consumption greater than 10% of the sample, a chi-square test (χ^2^) and the odds ratio (OR) were performed to check for possible differences both in terms of sex and competition level of sailors. The previous analysis was also applied to questions about their opinion on the consumption of SS in sport within the law, the consumption of SS and other questions related to motivation, place of purchase, or person who advises on the consumption of SS in those athletes who said they consume SS. Additionally, to analyze the differences in the consumption of SS based on the type of vessel, an analysis of variance (ANOVA) was carried out for the total consumption of supplements as well as for the consumption of the different categorizations and classification groups based on the level of scientific evidence established by the AIS (2019) [25]. A Holm–Bonferroni test was performed when a significant main effect was detected. Statistical significance was set at *p* < 0.05. The statistical analyses were performed using the Statistical Package for Social Sciences (version 18.0 for Mac, SPSSTM Inc, Chicago, IL, USA).

## 3. Results

### 3.1. Differences in the Consumption of Sport Supplements According to Sex

Although there was a greater proportion of men who declared to consume SS compared to women (54.8% vs. 45.5%), there were no significant differences in the prevalence of consumption between them (*p =* 0.209). Among those who consumed SS, higher consumption was observed on competition days (50.0% men and 57.1% women) with respect to the group that consumes them both in training and in competition (46.2% in men and 28.6% in women) and those who only consume them in training (3.8% in men and 14.3% in women).

Regarding the purpose of consumption, statistically significant differences were observed by sex (*p =* 0.017). Therefore, the proportion of men who consumed SS to improve their performance (65.4% vs. 28.6%), for improving their physical appearance (15.4% vs. 0%) or out of necessity (7.7% vs. 0%) was higher than women. On the contrary, the proportion of women who declared consumption to improve their health status (57.1% vs. 11.5%) or to prevent nutritional deficits (14.3% vs. 0%) was higher.

There were no statistically significant differences by sex (*p =* 0.327) regarding the place where they usually bought SS. Specialized stores (57.6%), pharmacies (21.2%), gyms (9.1%), supermarkets (6.1%), and nutritionists (6.1%) were the most frequent. In contrast, there were statistically significant differences by sex (*p =* 0.039) in terms of the person who advises them to consume SS. Therefore, men were advised more frequently than women by coaches (42.3% vs. 28.6%), teammates (23.1% vs. 14.3%), friends (19.2% vs. 0%), doctors (7.7% vs. 0%), and advertising (7.7% vs. 0%), whereas women did it through family mediation (42.9% vs. 3.8%) or nutritionists (14.3% vs. 0%).

Although SS consumption was higher in men, no differences were observed between men and women (5.00 ± 11.5 vs. 2.73 ± 3.2, *p =* 0.524; ES = 0.23) in terms of the number of SS consumed. In the analysis of the different groups of SS according to the level of scientific evidence, higher consumption was observed in men compared to women in all groups (A, B, C, and D), although no statistically significant differences were observed by sex (*p =* 0.390–0.788; ES = 0.10–0.31) (see Table 1).

### 3.2. Differences in the Consumption of Sports Supplements Depending on the Level of Competition

Concerning the consumption of SS, 70% of the sailors of international level declared to consume SS compared to 46.8% of those of national level, with the level of competition being a predisposing factor to consume these (OR = 1.38 [1.12−1.69]). Among those who consumed SS, higher consumption was observed on the days of competition (55.6% athletes of international level and 50.0% national level) with respect to the group that consumed them both in training and in competition (44.4% international level and 41.7% national level), or with respect to the group that consumed them only during training (0% international level and with 8.3% national level).

Regarding the purpose of consumption, no statistically significant differences were observed among sailors of different competitive levels (*p =* 0.225), although there was a greater proportion of athletes of international level whose motive was the improvement of performance (77.8% vs. 50.0%) or the improvement of their physical appearance (22.2% vs. 8.3%), while some athletes of the national level declared that their main motivation was to improve their state of health (29.2%), out of necessity (8.3%), or to prevent nutritional deficits (4.2%).

About the place where they usually bought the SS, no statistically significant differences were found in terms of competitive level (*p =* 0.831), although international athletes bought more frequently in specialized stores (44.4%), pharmacies (22.2% vs. 20.8%), gyms (11.1% vs. 8.3%), supermarkets (11.1% vs. 4.2%), or directly from nutritionists (11.1% vs. 4.2%), while those at the national level bought them more frequently in specialized stores (62.5%). With respect to the person who advises them to buy SS, athletes of international level were advised more frequently than those of national level by coaches (44.4% vs. 37.5%), teammates (33.3% vs. 16.7%), and doctors (11.1% vs. 4.2%), while those at national level did so through the mediation of the family (16.7% vs. 0%), friends (16.7% vs. 11.1%), nutritionists (4.2% vs. 0%), or advertising (4.2 % vs. 0%).

According to the competition level of the sailors, no differences were observed in the total number of SS consumed in sailors of international level with respect to those of national level (4.70 ± 3.65 vs. 4.31 ± 11.33; *p =* 0.916; ES = 0.04), with higher consumption of SS in Groups A and B in those of international level, while the consumption of the number of SS of Group C was higher in athletes of national level, although there were no statistically significant differences (*p* > 0.05). Regarding the consumption of supplements of Group D, none of the athletes of international level declared to consume them. As can be seen in Table 2, only a higher consumption of supplements of the sport foods category was observed in athletes of international level with respect to those of national level (2.00 ± 0.94 vs. 1.09 ± 1.03; *p =* 0.017; ES = 0.92).

### 3.3. Most Consumed Sports Supplements by Sex And Level of Competition

Regarding the most consumed SS (Table 3), no significant differences were observed between men and women, although men had a higher consumption of isotonic drinks (61.3% vs. 27.3%) and caffeine (29.0% vs. 9.1%), and women consumed more vitamin D (9.7% vs. 18.2%) and vitamin complexes (9.7% vs. 18.2%). It is worth noting the equality in the prevalence of consumption of bars (~ 65%) in both sexes.

Concerning the level of competition, the athletes of international level showed a higher non-significant consumption of supplements such as bars (80.0% vs. 59.4%), vitamin complexes (20.0% vs. 9.4%), and tyrosine (30.0% vs. 18.8%), although a significantly higher consumption of dextrose was observed (40.0% vs. 3.1%; *p =* 0.008), as well as a greater probability of consuming isotonic drinks (80.0% vs. 43.8%; OR = 1.41 [1.00–2.00]). On the other hand, the consumption of caffeine (25% vs. 20.0%) and vitamin D (12.5% vs. 10.0%) was slightly higher in athletes at the national level (see Table 3).

### 3.4. Differences in the Consumption of Sports Supplements in the Different Types of Boats

**Figure 1:**
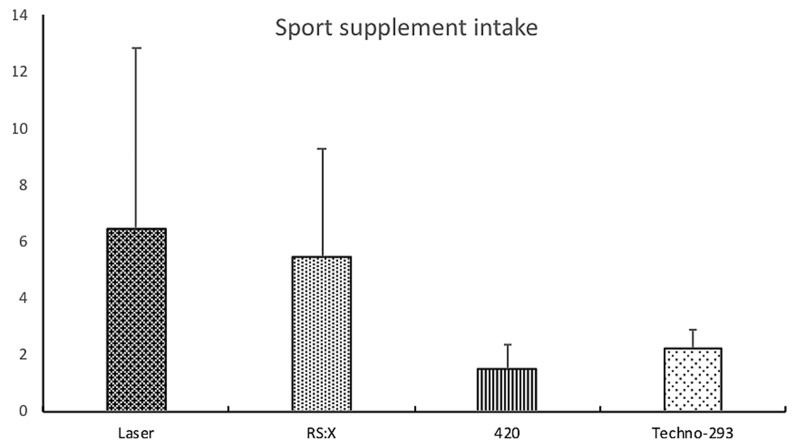
Intake of sports supplements in the different boat types.

Respecting to the total intake of SS according to the different vessel types, it was found that, although the average consumption was higher in the Laser class athletes (6.47 ± 16.39) compared to those of RS:X (5.44 ± 3.81), 420 (1.50 ± 0.84), and Techno-293 (2.50 ± 2.24), there were no statistically significant differences between classes (*p =* 0.658; see Figure 1). Regarding the analysis of the consumption of the different SS of the different groups according to the level of scientific evidence and the different categories, no statistically significant differences were detected in any group, and, only in the sports food category, there was a tendency to the significance of higher consumption in RS:X class athletes compared to Laser athletes (2.11 ± 0.93 vs. 1.00 ± 1.26, *p =* 0.082; see Table 4).

## 4. Discussion

The prevalence of SS consumption varies greatly between sports depending on different variables, among which the sport modality stands out. To this respect, current studies place the consumption of SS between 39% and 100% [21,23,24,28,29,30,31,32]. The data obtained in the present study are within the range described above, since 52.4% of the sample had consumed some SS on some occasion and 42.9% currently consumes it. These data are below the results of the GOAL study that examined 1138 German elite teenage athletes (14–18 years old; 91.1%) [34], although they are similar to the data reported in 536 young German elite athletes (55%) [35]. If we compare the results with a similar sample of 567 young non-elite Canadian athletes, a lower SS consumption can also be observed (98%) [36]. This may be due to the type of sport, although studies such as that carried out with 44 elite sailing athletes reported a greater use of SS, since 77% of these consumed it and 38% did it regularly (daily) [13]. Furthermore, another study conducted with 160 athletes from 30 sports, including sailing, reported consumption of 77% [37].

In addition to the sports modality, there are two variables that influence the prevalence of SS consumption: sex and the level of competition of the athlete. Therefore, the existing bibliography shows a higher consumption of SS in men vs. women [38], and an increase in their use in elite vs. amateur athletes and in those who compete internationally vs. nationally [21,24,27,39]. These statements are supported by the results of the present study in which there is a higher consumption of SS in men and international sailors compared to women and national sailors, respectively.

The obtained results also show that class 420 has the lowest SS consumption compared to the Laser, RS:X, and Techno-293 classes. This could be due to the fact that, in vessels with a double crew, as is the case of class 420, the performance requirements are more tactical and technical rather than physical and/or physiological, which is higher in boats with only one crew member [19,40]. Based on this, some studies claim that navigating in single-crew classes is the only factor that will significantly predict the more frequent use of SS [13].

The results show higher consumption of SS in the days of competition with respect to the rest of the options, regardless of sex or the level of competition. This behavior may be driven by the fact that nutrition and hydration can be compromised during competition [20], due to the physical demand and limited access to liquid and solid foods [16]. In fact, when observing the most consumed SS (see Table 3), isotonic bars, and drinks are among the most consumed supplements. Both supplements are important for the replenishment of nutrients and hydration during competition periods, providing energy and nutrients when it is not practical to consume daily foods due to the limitation of the boat in the case of the bars, and seeking to counteract the high sweating rates generated by the typical weather conditions of this sport in the case of sports drinks [41,42]. In addition, the use of these two SS can prevent the deterioration of endurance, strength, blood volume, and cognitive function during competition [43]. It should also be added that, among the most consumed SS, caffeine and dextrose also stand out, which are also often used during competition [1,44].

The purpose of SS consumption is addressed in the existing literature since it gives a real perspective of its use. Therefore, it seems that the purpose of consumption, as shown by the results of this study, is differentiated by sex, with the improvement of performance or physical appearance being the most common purpose in men and health or prevention of nutritional deficits in women [22,30,45]. On the other hand, and as expected, the most popular option in competition levels was the improvement of performance in both cases, although with a greater proportion in international than in national competitions (77.8% vs. 50.0%). In general, regardless of the studied variables, one of the contributions of this research is that, in light sailing athletes, the main motivations for the consumption of SS are in line with those reported in the existing literature among elite athletes, being performance and health the most frequent motivations [39].

The obtained results regarding the place where the SS are bought contradict the current data since the current trend is online shopping [23,28]. The participants of the present study, regardless of sex and the level of competition, are more inclined to buy in specialized stores and pharmacies mainly, where they can also find more professional advice compare to online shopping, which decreases the possibilities of purchasing lower-quality or illegal products, with lesser guarantees due to the absence of specific legislation in Europe or other countries, contributing to misleading advertising [39,46].

This topic is related to the person or source that generates or advises the use of SS. Thus, it would be ideal that the advice was in the hands of experts in this field such as nutritionists or sports doctors (who, on the contrary, are the least chosen by the sample), who seeks more advice in coaches, colleagues from the team, and family or friends, as is reported in numerous studies [22,23,28,29,30,47,48]. This may explain why some of the participants, regardless of sex or level of competition, consume SS from Group C (see Table 1, Table 2 and Table 4), a group of supplements without scientifically proven ergogenic effects [25], which can also be associated with possible negative effects, causing a deterioration of sports performance and/or adverse effects on the health of the athlete [49]. This can generate an inappropriate use of SS, not only because of their low safety, efficacy, and legality but also because of their use in unfavorable or disapproved situations [39,49,50,51].

The results found in the analysis of the different groups of SS classified according to the level of scientific evidence established by the AIS (2019) show that there were no statistically significant differences according to sex, level of competition, or type of vessel in the sample. It should be noted in this regard that, of the seven most consumed SS, five belong to Group A, one to Group B and one to Group C (see Table 3). In general, it is also observed that the participants, regardless of the variables with which they are related, have a higher consumption of SS of Group C than of Group B, with those of Group A being the most consumed (see Table 1, Table 2 and Table 4).

In response to the high demands for isometric strength and resistance that sailors must face [11,12], as well as the high % VO_2max_ they reach as the speed increases [9,14], those athletes who consume caffeine (20% sailors of international level and 25% of those of national level) can benefit from the use of this supplement. Since the molecular form resembles that of adenosine, caffeine blocks adenosine receptors, activating the central nervous system, which decreases the subjective perception of effort, while enhancing glycolytic metabolism, neuromuscular recruitment, and the bioavailability of calcium in myoplasm [52]; moreover, previous studies have demonstrated its ergogenic effect on the improvement of muscle strength [53] and in the face of high-intensity efforts [54].

In addition to caffeine, supplements with beet juice (nitrate-rich nutritional source) increases nitric oxide levels. Through nitric oxide, nitrate supplements improve vasodilation, irrigation, and muscle contraction strength [55], with beet juice being a supplement that has demonstrated its ergogenic effect on both endurance capacity [56] and on strength and high-intensity efforts [57].

If we look at the quasi-isometric actions [8], which could be a stimulus to increase intramuscular pressure, resulting in a capillary collapse and an increase in glycolysis due to lower bioavailability of oxygen [58], supplementation with β-alanine and sodium bicarbonate could improve the performance of sailors. Therefore, supplementation with β-alanine increases levels of muscle carnosine [59], which acts as a shuttle transporting calcium from the sarcoplasmic reticulum to the myoplasm, capturing ions and transporting them to the outside of the muscle cell [60]; thus, it is considered an ergogenic supplement in both resistance and high-intensity efforts [61]. On the other hand, supplements with sodium bicarbonate increase serum levels, causing an alkalosis that favors the diffusion of H^+^ produced by glycolysis due to an increase in the concentration gradient and potentiation of glycolysis [62], improving the performance of both endurance strength [63] and high-intensity efforts [64].

In addition, the characteristics of the competitions, the boats, and the weather conditions in dinghy sailing can also lead to the inclusion of supplements of the sports foods category of Group A, such as drinks and sports gels, sports bars, liquid foods or whey protein. The inclusion of sports drinks is essential to restore the sailor’s hydroelectrolytic balance [65] and, thus, to alleviate one of the most frequent problems during sailing competitions, i.e., dehydration, and the consequent deterioration of performance and health of the sailor [16,17,18,43]. In addition, these drinks should include carbohydrates, which, added to their electrolytes, prevent the deterioration of endurance, strength, blood volume, and cognitive function in competitions of this duration [43,66]. The inclusion of sports foods such as bars or liquid meals can help the maintenance and replenishment of muscle glycogen, essential for maintaining performance and important in the light sail that includes multiple competition sessions on the same day or on successive days [43,65]. Due to the long duration of some of these competitions (sometimes longer than 5 h), protein and carbohydrate co-management is necessary to avoid prolonged periods of muscle protein degradation, especially when the competition lasts several successive days, helping in turn to reduce the muscle damage produced and to improve post-exercise recovery [43,65,66].

The scarce existing bibliography shows a general rejection of the use of prohibited substances included in doping. In this sense, the results obtained by some studies show that doping is not usually present in sailing sports. Its casuistry is so low that, in 2011, the World Anti-Doping Agency found only four cases [13]. These data are corroborated by the results of the present study, in which a very low consumption of Group D supplements can be observed, which is the group of prohibited supplements or with a risk of contamination by positive substances in doping [25]. In this regard, the consumption of SS of Group C by the sample may cause “masked doping”, that is, the consumption of supplements that may include undeclared substances unknown to the consumer [21,39,42,67]. Apart from resulting in a doping penalty, this can also cause serious health problems in the athlete [49].

Different studies associate the lack of sailors’ knowledge of sports sciences and their sports environment with the inappropriate use of nutritional and water aspects during competitions [16,17,43]. In this regard, determining the hydric and nutritional requirements in sailing sports, currently not established, and improving knowledge is important for improving the performance of the athlete and ensuring his/her good health.

Due to the narrow margin in sports between success and defeat, and in search of an improvement in performance, many athletes resort to the consumption of SS [44]. Compared to factors such as genetics, training, or the athlete’s own diet, SS are, and should be, a complementary part to the proper planning of the athlete. However, contradictorily, too much emphasis is placed on the consumption of these [39]. SS must be considered as a complement to the habitually consumed diet, which is consumed with the aim of achieving specific health and/or performance benefits [21]. The use of SS is mostly targeted to improve (i) the management of micronutrient deficiencies, (ii) the supply of convenient forms of energy and macronutrients, (iii) provision of direct benefits to performance, or (iv) indirect benefits such as supporting intense training regimens [21]. They are rarely effective outside of these conditions, and it is not justified in the case of young athletes who can have significant performance gains through maturation, sports experience, or the development of a sports nutrition plan [50]. Standardization and categorization of SS are essential for their control in both amateur athletes and elite athletes [39]. The risks of using SS, their effective use, and optimizing the intake of nutrients from food to minimize the use of these are necessary fields of research and education [27].

## 5. Conclusions

The prevalence of SS consumption in sailors in this study was ~50%, with SS consumption being higher in men compared to women and in sailors of international level compared to those of national level. By classes, it was proved that class 420 had the lowest consumption, while the intake of SS was higher during competition days, regardless of sex or level of competition. The purpose of SS consumption in men was oriented to performance, while in women, the main motivations were health and prevention of nutritional deficits. The results obtained from the analysis of the different groups of SS classified according to the level of scientific evidence established by the AIS (2019) showed that there were no statistically significant differences according to sex, level of competition, or type of vessel in the sample.

## Figures and Tables

**Table 1:** Consumption of sports supplements of the different groups and categories established by the AIS (2019) in male and female sailors.

AIS Group	Type of Supplement	Males	Females	*p*-Value	ES
**Group A**	Sport foods	1.45 ± 1.12	0.91 ± 0.83	0.151	0.52
	Medical supplements	0.32 ± 0.87	0.45 ± 0.69	0.653	0.16
	Performance supplements	0.49 ± 1.15	0.18 ± 0.40	0.402	0.31
	Total group A	2.26 ± 2.54	1.55 ± 1.57	0.390	0.31
**Group B**	Sick pack	0.23 ± 0.50	0.27 ± 0.47	0.786	0.08
	Antioxidants	0.29 ± 0.64	0.36 ± 0.67	0.750	0.11
	Amino acids	0.16 ± 0.64	0.00 ± 0.00	0.410	0.30
	Others	0.16 ± 0.90	0.00 ± 0.00	0.558	0.21
	Total group B	0.84 ± 2.37	0.63 ± 1.12	0.788	0.10
**Group C**	Total group C	1.87 ± 6.77	0.55 ± 0.93	0.525	0.23
**Group D**	Total group D	0.03 ± 0.18	0.00	0.558	0.20

Data expressed as median ± standard deviation.

**Table 2:** Consumption of sports supplements of the different groups and categorization established by the AIS (2019) in national and international sailors.

AIS Group	Type of Supplement	National	International	*p*-Value	ES
**Group A**	Sport foods	1.09 ± 1.03	2.00 ± 0.94	0.017 *	0.92
	Medical supplements	0.38 ± 0.87	0.30 ± 0.67	0.804	0.10
	Performance supplements	0.47 ± 1.13	0.20 ± 0.42	0.471	0.28
	Total group A	1.94 ± 2.55	2.50 ± 1.43	0.512	0.25
**Group B**	Sick pack	0.22 ± 0.49	0.30 ± 0.48	0.649	0.17
	Amino acids	0.09 ± 0.53	0.20 ± 0.63	0.600	0.20
	Antioxidants	0.28 ± 0.63	0.40 ± 0.70	0.617	0.19
	Others	0.16 ± 0.88	0.00	0.582	0.22
	Total group B	0.75 ± 2.24	0.90 ± 1.66	0.847	0.07
**Group C**	Total group C	1.59 ± 6.69	1.30 ± 1.16	0.892	0.05
**Group D**	Total group D	0.03 ± 0.18	0.00	0.582	0.20

Data expressed as median ± standard deviation. * Statistical difference in the consumption of sailors of international vs. national levels (*p* < 0.05).

**Table 3:** Sports supplement consumption according to sex and competitive level.

AIS Group	Supplement	Sex				Competitive Level			
		Males (*n =* 31)	Females (*n =* 11)	*p*-Value	OR	National (*n =* 32)	International (*n =* 10)	*p*-Value	OR
**Group A**	Bars	64.5% (20)	63.6% (7)	1.00	1.03 [0.36–2.95]	59.4% (19)	80.0% (8)	0.286	1.23 [0.90–1.69]
	Isotonic drink	61.3% (19)	27.3% (3)	0.081	2.93 [0.90–9.55]	43.8% (14)	80.0% (8)	0.071	1.41 [1.00–2.00]
	Vitamin D	9.7% (3)	18.2% (2)	0.593	0.61 [0.18–2.05]	12.5% (4)	10.0% (1)	1.00	0.95 [0.59–1.52]
	Vitamin complex	9.7% (3)	18.2% (2)	0.593	0.61 [0.18–2.05]	9.4% (3)	20.0% (2)	0.577	1.31 [0.63–2.73]
	Caffeine	29.0% (9)	9.1% (1)	0.245	3.13 [0.45–21.51]	25.0% (8)	20.0% (2)	1.00	0.94 [0.65–1.36]
**Group B**	Tyrosine	19.4% (6)	27.3% (3)	0.67	0.73 [0.24–2.19]	18.8% (6)	30.0% (3)	0.660	1.18 [0.72–1.94]
**Group C**	Dextrose	12.9% (4)	9.1% (1)	1.00	1.35 [0.22–8.44]	3.1% (1)	40.0% (4)	0.008 *	4.19 [0.72–24.32]

* Statistical difference in consumption between groups (*p* < 0.05); OR: odds ratio.

**Table 4:** Consumption of sports supplements of the different groups and categorization established by the Australian Institute of Sport (AIS) 2019 in sailors of different boats.

AIS Group	Type of Supplement	Laser	RS:X	420	Techno-293	*p*-Value
**Group A**	Sport foods	1.00 ± 1.26	2.11 ± 0.93	1.17 ± 0.98	1.16 ± 0.72	0.079
	Medical supplements	0.53 ± 1.13	0.44 ± 0.73	0.00 ± 0.00	0.25 ± 0.62	0.561
	Performance Supplements	0.73 ± 1.58	0.50 ± 0.17	0.00 ± 0.00	0.25 ± 0.45	0.427
	Total group A	2.27 ± 3.51	2.89 ± 1.54	1.17 ± 0.98	1.67 ± 1.07	0.494
**Group B**	Sick pack	0.20 ± 0.56	0.44 ± 0.53	0.17 ± 0.41	0.17 ± 0.39	0.564
	Amino acids	0.20 ± 0.67	0.22 ± 0.67	0.00 ± 0.00	0.00 ± 0.00	0.701
	Antioxidants	0.27 ± 0.70	0.67 ± 0.86	0.17 ± 0.41	0.17 ± 0.39	0.300
	Others	0.33 ± 1.29	0.00 ± 0.00	0.00 ± 0.00	0.00 ± 0.00	0.631
	Total group B	1.00 ± 3.14	1.33 ± 1.80	0.33 ± 0.82	0.33 ± 0.78	0.675
**Group C**	Total group C	3.13 ± 9.70	1.22 ± 0.97	0.00 ± 0.00	0.50 ± 0.90	0.601
**Group D**	Total group D	0.067 ± 0.26	0.00 ± 0.00	0.00 ± 0.00	0.00 ± 0.00	0.631

Data expressed as Medium ± standard deviation.
